# Granulomatous Reaction to Lip Filler in a Patient with Ankylosing Spondylitis: A Case Report

**DOI:** 10.7759/cureus.71032

**Published:** 2024-10-07

**Authors:** Angelique Ruml, Ibeth P Caceres, Shannon Sayyadioskoie, Jay R Patel, Zeena Nawas

**Affiliations:** 1 Dermatology, Baylor College of Medicine, Houston, USA

**Keywords:** ankylosing spondylitis, autoimmune disease, case report, dermal filler, granulomatous reaction, hyaluronic acid, hyaluronidase

## Abstract

The popularity of nonsurgical, minimally invasive cosmetic treatments has increased drastically in recent years, with lip augmentation being one of the most common. Dermatologists play an important role in the delivery of safe, evidence-based options for lip augmentation, including lip filler. Many types of filler are available to patients, and their safety profiles have been studied extensively. Adverse effects, if they do occur, are generally mild and self-limited; however, fewer data exist describing the adverse effects of fillers, particularly hyaluronic acid (HA) dermal fillers, in patients with a history of autoimmune disease. This report presents a case of a granulomatous delayed hypersensitivity reaction to HA filler in a patient with ankylosing spondylitis, which was subsequently successfully treated with hyaluronidase. As complications of cosmetic procedures affect patients' quality of life, this case highlights the importance of recognizing similar reactions to HA filler and the efficacy of hyaluronidase as a treatment option when they occur.

## Introduction

According to the American Society of Plastic Surgeons, lip augmentation was the fifth most common cosmetic minimally invasive procedure in 2022, with 1.4 million procedures performed in 2022 alone [1]. Dermal fillers, also known as facial fillers, are soft, gel-like substances. They can be used for scars, volume deficiency, and facial augmentation [1]. Since the approval of the first dermal filler by the United States Food and Drug Administration (FDA) in 1981, there are now four groups of approved fillers: hyaluronic acid (HA), polymethyl methacrylate, poly-L-lactic acid, and calcium hydroxylapatite [2]. Of the many options available, HA fillers are the most often used, particularly for lip augmentation [2]. Given that the use of lip fillers continues to grow at a rapid rate, with a two-fold increase since 2019, it is essential to give careful consideration to appropriate patient selection to minimize the incidence of complications [1]. Adverse events associated with HA filler treatment are generally mild, self-limiting, and reversible, such as injection site reactions, including edema, pain, erythema, itching, and ecchymosis [2]. However, more serious adverse events ranging from infections to blindness and skin necrosis have been described [2].

Further, conditions requiring physician discretion or contraindicating the use of dermal fillers include active localized and generalized infections, hypersensitivity to filler, mixed connective tissue disease, and patients with skin atrophy secondary to chronic corticosteroid use, among many others [3]. Although it is known that the use of dermal filler in patients with autoimmune conditions such as rheumatoid arthritis and inflammatory bowel disease warrants caution due to increased immune reactivity, less is known regarding the safety of dermal filler in patients with other autoimmune diseases, notably ankylosing spondylitis [3]. Here, we describe a case of biopsy-proven granulomatous reaction to HA lip filler in a patient with ankylosing spondylitis.

## Case presentation

A 53-year-old female with a two-year history of ankylosing spondylitis, well-controlled on etanercept, initially presented to the otolaryngology clinic with a three-month history of painful subcutaneous nodules affecting her lips and jaw.

The patient denied dysphagia, otalgia, hemoptysis, cough, fever, chills, and recent weight loss. Further questioning revealed that seven months prior to presentation, the patient received Juvederm Ultra injections to the upper and lower lips and Sculptra injections to the midface at a medical spa. Three months prior to presentation, the patient developed a firm, nonpruritic, painless nodule along the right mandible, which progressively worsened to what she was experiencing at the time. She was given a dose of piperacillin/tazobactam and discharged with 10 days of amoxicillin/clavulanic acid and a five-day course of prednisone 20 mg twice daily. Symptoms improved slightly but persisted. An ultrasound of the head and neck from an outside facility showed three hypoechoic areas within the lower right and left cheek subcutaneous tissues, representing phlegmons. A maxillofacial CT without contrast showed bilateral mandibular soft tissue swelling and fatty stranding, most consistent with cellulitis. No abscess, acute osseous process, or evidence of acute sinusitis was noted. At this point, she was referred to the dermatology clinic.

**Figure 1:**
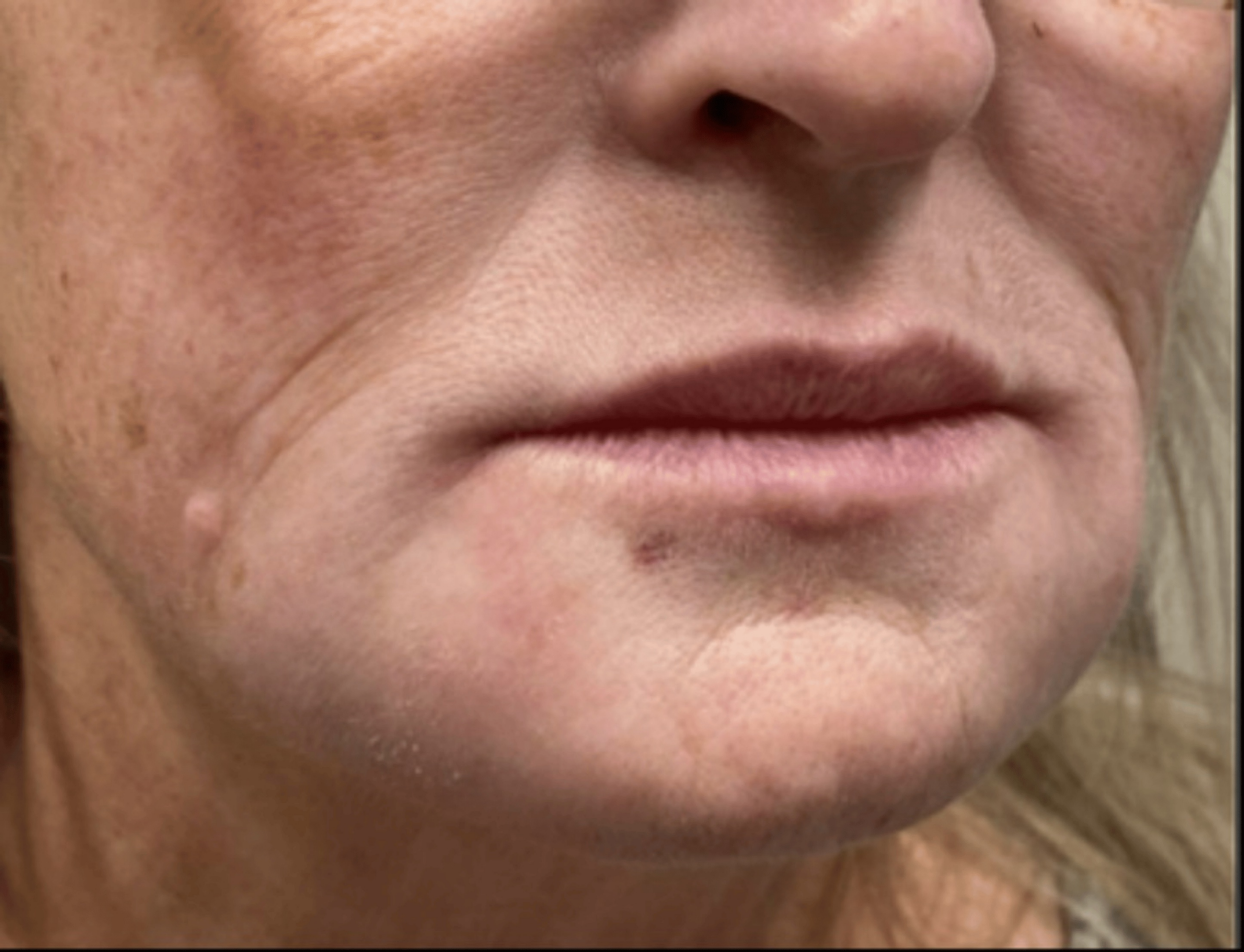
Clinical presentation demonstrating erythematous to skin-colored papules and nodules on bilateral jaws and lips

**Figure 2:**
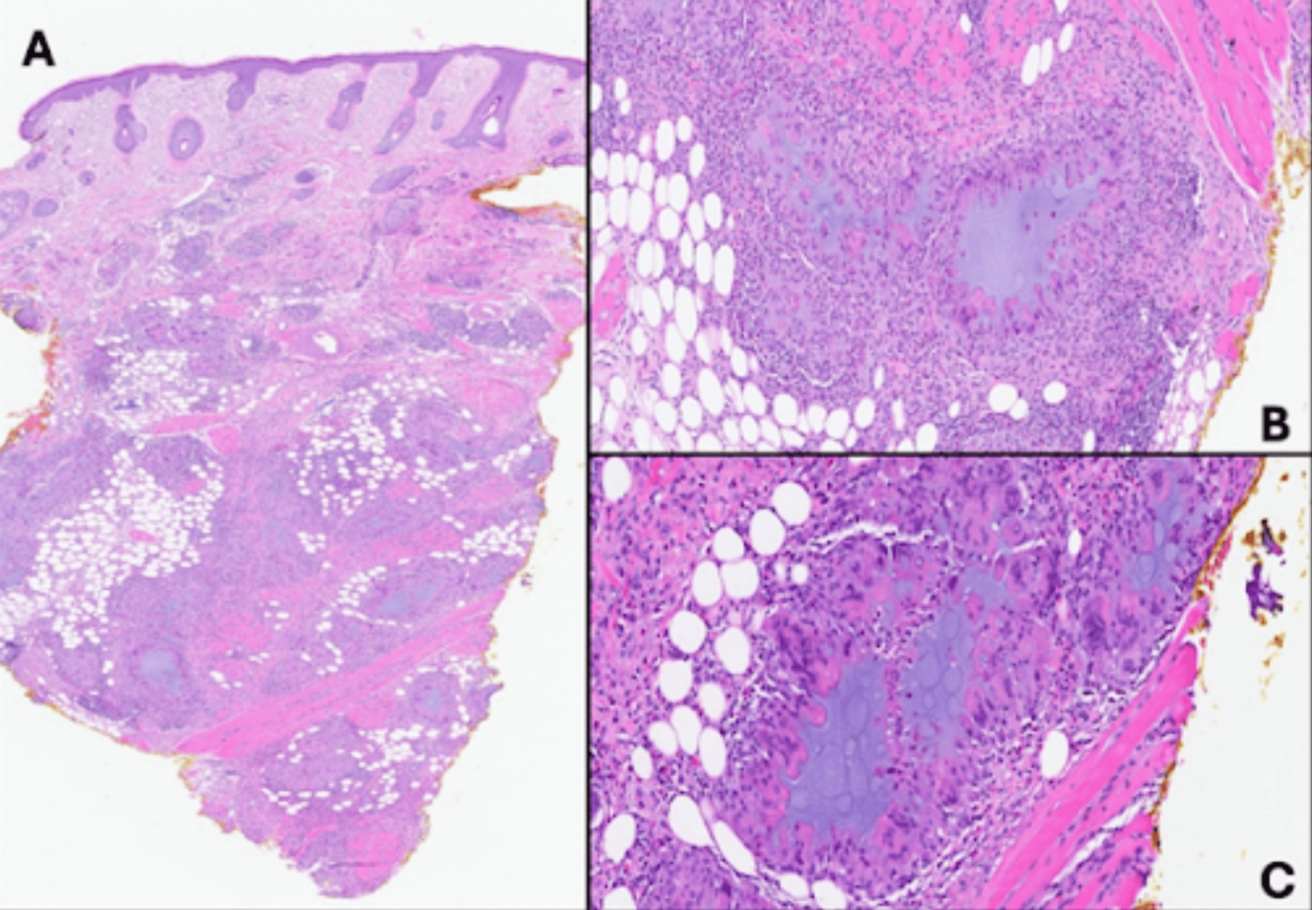
Low-power hematoxylin and eosin stain showing an inflammatory process located in the dermis and subcutis (A). Higher magnification demonstrating violaceous, homogenous, amorphous material surrounded by histiocytes and giant cells (B,C)

On exam, the patient had a single 1-2 cm firm tender subcutaneous nodule along the left anterior mandible, numerous firm non-tender nodules on the lower cutaneous lip and marionette lines, and a few subcentimeter firm nodules on the right anterior mandible without overlying erythema or skin changes (Figure 1). A punch biopsy of a lesion on the right chin was taken at that time and stained with hematoxylin and eosin. Histological findings revealed a robust mixed inflammatory infiltrate containing giant cells and grayish material consistent with HA (Figure 2).

**Figure 3:**
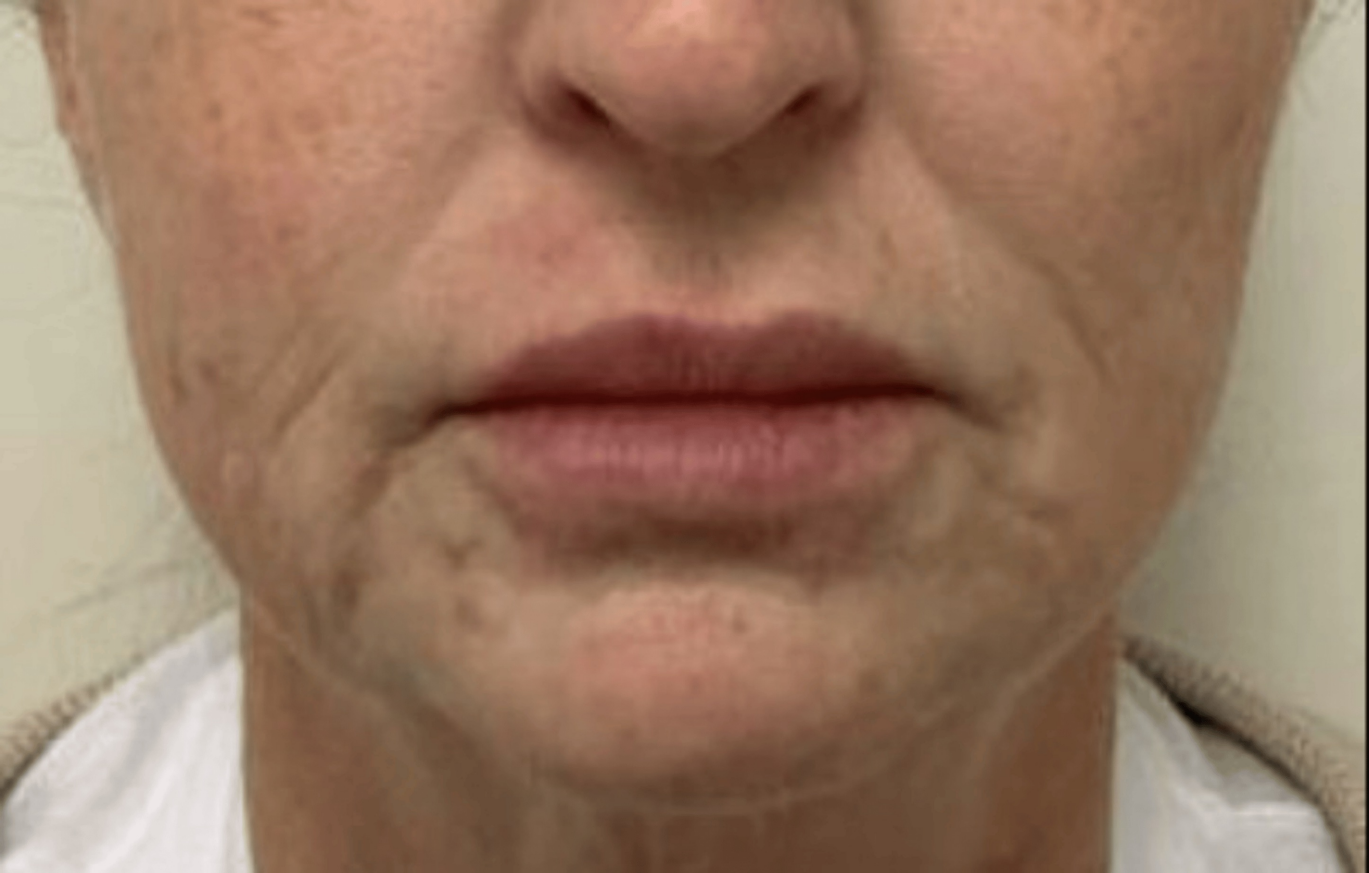
Resolution of erythematous and skin-colored papules and nodules on bilateral jaws and lips

While the patient's history and biopsy results were suspicious for a delayed sensitivity reaction to Juvederm injections, due to continued worsening of symptoms and immunosuppression secondary to etanercept, additional biopsies were taken for cultures to rule out a primary infection, which ultimately showed no growth. Based on clinical history, classic histologic findings, and negative cultures, a diagnosis of granulomatous delayed hypersensitivity reaction (DHR) to HA filler was made. Interestingly, the patient reported previous neurotoxin Botox® injections and lip filler approximately five years ago. No skin changes were noted at that time. Since symptoms developed, the patient has not undergone any other cosmetic treatments. Hyaluronidase 1 mL was injected into the affected areas of the mucosal and cutaneous upper and lower lips and bilateral anterior mandible. Three weeks later, the patient returned to the clinic with dramatic improvement. A second treatment of hyaluronidase 1 mL in the same areas was performed, demonstrating clearance of these nodules three weeks later (Figure 3). The patient was counseled that, given her rheumatologic history and recent reaction, she should forgo further injections with filler.

## Discussion

The field of non-surgical aesthetic procedures has witnessed a significant surge in demand in recent years, with neuromodulators, skin treatments, and dermal fillers contributing significantly to the industry's growth [4]. HA-based dermal filler for use in lip augmentation is no exception to these trends. The ideal dermal filler should be natural-appearing, long-lasting, consistent, and have predictable results along with minimal risk of complications. Although the perfect filler does not exist, the number of injectable skin fillers available on the market continues to increase with advances in technology [5]. Despite the growing safety of fillers, adverse events such as injection site reactions, ecchymosis, infections, surface irregularity, and the Tyndall effect, among others, can still occur [2].

DHR, also known as Type IV hypersensitivity reactions, are T-cell-mediated immune responses to exogenous or autoantigens in sensitized individuals [6]. Three variants of DHR exist, including contact, tuberculin, and granulomatous reactions [6]. All DHR variants take over 12 hours to develop, with a maximal response for granulomatous reactions occurring between 21 and 28 days [6]. DHR involves both T-cells and antigen-presenting cells, which stimulate cytokines, producing a local inflammatory response through the recruitment of T-cells and macrophages [6]. However, the mechanism by which HA fillers cause DHR is not fully elucidated. Also, most granulomatous DHRs develop around primary injection sites, but granulomas may form at distant sites due to migration of the dermal filler material [7]. This may occur on any body part through vascular or lymphatic transport, along anatomical structures, by gravity, and along with inflammatory reactions through transport by macrophages [7]. Our patient only had filler injected in her mucosal lip, but the fillers were found in the mucosal lip, marionette line, and along the anterior mandible. The incidence of foreign body granuloma secondary to HA filler is 0.02%-0.4% [8]. Studies have implicated injection technique, intrinsic filler properties, history of infections, and trauma at injection sites as possible risk factors [9].

HA fillers consist of the cross-linker, 1,4-butanediol diglycidyl ether, and high molecular weight HA [8]. Further, HA fillers may contain impurities such as stainless steel, aluminum, and silicone oil secondary to the manufacturing process. Although these impurities have been shown to cause DHR in the literature, currently, no studies directly show that these impurities lead to DHR in the context of dermal fillers. Juvederm Ultra, the dermal filler administered to this patient, contains a bacteria-derived HA, which is identical across species, reducing its recognition as a foreign substance [8].

Ankylosing spondylitis is a chronic, autoinflammatory condition within the family of spondyloarthropathies. Active ankylosing spondylitis is characterized by inflammation at the sacroiliac joint and enthesitis, which can lead to bone fusion and long-term severe chronic pain [10]. The pathogenesis is unclear, but evidence suggests there is dysregulation between the HLA-B27 protein complex and innate immune cells, causing autoreactivity [11,12]. These immune cells (notably macrophages, which secrete pro-inflammatory factors such as interleukin-23) contribute to a state of active inflammation, acting as a noninfectious trigger of granuloma formation [13]. Non-steroidal anti-inflammatory drugs and physical therapy are considered first-line treatments, but patients with moderate to severe disease need biologic therapy [10]. While there are no reported cases of granulomatous reaction to filler in ankylosing spondylitis to our knowledge, its incidence in patients with autoimmune disease is well documented. As a result of her immune system's misidentification of self-antigens, the patient has inflammatory dysregulation at baseline compared to the general population. It is likely that this and the degree of immunosuppression associated with her etanercept therapy contributed to her significant foreign body reaction. While tumor necrosis factor-alpha inhibitors are often used as treatment of granulomatous disease, they have also been linked to granulomatous drug reactions.

More research is needed to characterize the difference between the granulomatous reaction to filler in patients with concomitant autoimmune diseases and those without. It has been reported that their use in patients with autoimmune disease, including those treated with biologic agents, was associated with a risk of mild, transient site reactions, which healthy patients may also experience [2,14]. However, this depends on the degree of control of the autoimmune condition and the presence of disease-modifying therapy.

The main goal of filler use is always to prevent complications; however, this is not always possible. Treatment will depend on the specific adverse event. Evidence has shown that granulomatous reactions to HA fillers can be treated with hyaluronidase at a dose of 150 units/mL, which was used in our patient with marked clinical improvement [12]. While the patient did not develop any inflammatory lesions, intralesional injections with triamcinolone or fluorouracil could have been considered if she had not improved.

## Conclusions

The use of HA fillers has increased, and it is a common minimally invasive cosmetic procedure. There is scarce information relating specifically to the effectiveness and safety of aesthetic procedures in ankylosing spondylitis. With the rise in accessibility of these products to the general public, it is crucial to counsel patients with autoimmune conditions about the potential adverse effects of HA fillers in exacerbating inflammatory responses, particularly when the patient is on an immunosuppressive regimen such as in our patient. While the treatment for adverse events depends on the specific complication, we present a case of delayed granulomatous reaction successfully treated with two rounds of hyaluronidase injections. Overall, clinical vigilance and thorough history-taking are warranted to minimize adverse outcomes and maximize patient satisfaction.
